# Genetic Association between ACE2 (rs2285666 and rs2074192) and TMPRSS2 (rs12329760 and rs2070788) Polymorphisms with Post-COVID Symptoms in Previously Hospitalized COVID-19 Survivors

**DOI:** 10.3390/genes13111935

**Published:** 2022-10-24

**Authors:** César Fernández-de-las-Peñas, Lars Arendt-Nielsen, Gema Díaz-Gil, Francisco Gómez-Esquer, Antonio Gil-Crujera, Stella M. Gómez-Sánchez, Silvia Ambite-Quesada, María A. Palomar-Gallego, Oscar J. Pellicer-Valero, Rocco Giordano

**Affiliations:** 1 Department of Physical Therapy, Occupational Therapy, Rehabilitation and Physical Medicine, Universidad Rey Juan Carlos, 28922 Alcorcón, Spain; 2 Center for Neuroplasticity and Pain (CNAP), SMI, Department of Health Science and Technology, Faculty of Medicine, Aalborg University, DK-9220 Aalborg, Denmark; 3 Department of Medical Gastroenterology, Mech-Sense, Aalborg University Hospital, DK-9000 Aalborg, Denmark; 4 Research Group GAMDES, Department of Basic Health Sciences, Universidad Rey Juan Carlos (URJC), 28922 Alcorcón, Spain; 5 Intelligent Data Analysis Laboratory, Department of Electronic Engineering, ETSE (Engineering School), Universitat de València, 46100 Valencia, Spain

**Keywords:** single nucleotide polymorphism, ACE2, TMPRSS2, post-COVID, long-COVID

## Abstract

The aim of the study was to identify the association between four selected COVID-19 polymorphisms of ACE2 and TMPRSS2 receptors genes with the presence of long-COVID symptomatology in COVID-19 survivors. These genes were selected as they associate with the entry of the SARS-CoV-2 virus into the cells, so polymorphisms could be important for the prognoses of long-COVID symptoms. Two hundred and ninety-three (n = 293, 49.5% female, mean age: 55.6 ± 12.9 years) individuals who had been previously hospitalized due to COVID-19 were included. Three potential genotypes of the following single nucleotide polymorphisms (SNPs) were obtained from non-stimulated saliva samples of participants: ACE2 (rs2285666), ACE2 (rs2074192), TMPRSS2 (rs12329760), TMPRSS2 (rs2070788). Participants were asked to self-report the presence of any post-COVID defined as a symptom that started no later than one month after SARS-CoV-2 acute infection and whether the symptom persisted at the time of the study. At the time of the study (mean: 17.8, SD: 5.2 months after hospital discharge), 87.7% patients reported at least one symptom. Fatigue (62.8%), pain (39.9%) or memory loss (32.1%) were the most prevalent post-COVID symptoms. Overall, no differences in long-COVID symptoms were dependent on ACE2 rs2285666, ACE2 rs2074192, TMPRSS2 rs12329760, or TMPRSS2 rs2070788 genotypes. The four SNPs assessed, albeit previously associated with COVID-19 severity, do not predispose for developing long-COVID symptoms in people who were previously hospitalized due to COVID-19 during the first wave of the pandemic.

## 1. Introduction

The first cases of coronavirus disease, 2019 (COVID-19) were registered in Wuhan (China) in December 2019. The identification of the severe acute respiratory syndrome coronavirus 2 (SARS-CoV-2) virus, the agent responsible of COVID-19, was possible due to the analysis of DNA samples from lower respiratory tracts of infected individuals [1]. Sequencing studies of the SARS-CoV2 virus have identified 79% homology of this virus with its predecessor, the SARS-CoV-1, and also approximately 50% with Middle East respiratory syndrome coronavirus (MERS-CoV) [1]. Several studies have focused on the specific viral mechanisms of infection pointing to a shared entry pathway with previous coronaviruses, suggesting the involvement of surface receptor for S1 of the angiotensin-converting enzyme 2 (ACE2) and transmembrane protease serine-2 (TMPRSS2) receptor in COVID-19 [2].

Among different genes potentially involved in COVID-19, the association between the polymorphisms of renin-angiotensin-aldosterone system-related genes, i.e., ACE2 and TMPRSS2 and the severity of COVID-19 disease have been the most investigated [3]. In fact, ACE2 rs2285666 and TMPRSS2 rs12329760 single nucleotide polymorphisms (SNPs) have been investigated in several studies, although their results are controversial. Some studies have correlated the T (A) allele of the ACE2 rs2285666 [4] and of TMPRSS2 rs12329760 [5] SNPs with lower severity of COVID-19 in an Indian population. These data have been also replicated in a multicountry study [6] and confirmed by the meta-analysis by Dobrijevic et al. concluding that T alleles of ACE2 rs2285666 (OR 0.512, 95% CI 0.331–0.793) and TMPRSS2 rs12329760 (OR 0.734, 95% CI 0.560–0.960) SNPs were associated with lower risk of developing severe COVID-19 [7]. Another meta-analysis has found that TMPRSS2 rs12329760 was not associated with more severe COVID-19 manifestations, whereas ACE2 rs2285666 and ACE2 rs2074192 SNPs were associated with more severe COVID-19 manifestations by genotype but not by allele [8].

On the other hand, other studies have observed different results. Martínez-Gómez et al. have recently identified that the T allele of ACE2 rs2285666 was associated with higher risk for critical outcomes (OR 1.89, 95% CI 1.06–3.35) of COVID-19, especially for men [9]. Wulandari et al. observed that the TMPRSS2 rs12329760 was associated with a higher viral load but not with a more severe acute condition [10]. The meta-analysis by Saengsiwaritt et al. revealed an association between the C allele of the TMPRSS2 rs12329760 (OR 1.32, 95% CI 1.01–1.73) and the T allele of ACE2 rs2285666 (OR 2.14, 95% CI 1.26–3.66) with a higher risk of severe COVID-19 [11]. Current evidence suggests a clear influence of these polymorphisms in COVID-19, but makes more complex the understating of their association with the pathophysiology of the infection.

Even though ACE2 and TMPRSS2 receptors are highly expressed in the lungs and respiratory tract, explaining the tropism for SARS-CoV-2, COVID-19 patients reported disorders in other systems as these receptors are also detected in several other tissues and cells, e.g., macrophages, cardiomyocytes, muscle cells, renal tubular cells, and neurons [12]. This multisystemic presence of ACE2 or TMPRSS2 receptors explains the plethora of symptoms that these patients can exhibit during the acute phase of the infection [13].

Importantly, the COVID-19 pandemic had not only caused millions of acute cases and deaths, but is now making healthcare professionals face a second outbreak, characterized by the presence of symptoms after the acute phase of the infection in people previously infected by SARS-CoV-2, i.e., long-COVID [14] or post-COVID-19 condition [15]. Current meta-analyses support that around 30–50% of subjects who had survived SARS-CoV-2 infection develop persistent symptoms lasting at least one-year after [16,17,18]. More than 50 post-COVID symptoms have been described [19], those affecting the respiratory, (e.g., fatigue, dyspnea) and neurological (e.g., brain fog, memory loss, headache) systems being the most frequent [20]. Different studies have tried to identify risk factors associated with the development of post-COVID symptoms [21,22]. To date, female sex is the only factor which has been clearly associated with a higher risk of suffering from post-COVID [21,22]. Other factors, including the severity of the disease, previous co-morbidies or pre-infection health status have been identified in single studies as potential risk factors, but their role is not still conclusive [21,22]. Two previous reviews identified that post-COVID symptoms were not associated with COVID-19 severity [21,22]; however, a recent meta-analysis has found that higher severity of the disease at the acute phase of infection is associated with a higher risk of exhibiting post-COVID respiratory symptomatology (OR 1.66, 95%CI 1.03–2.68) [23]. Accordingly, since ACE2 and TMPRSS2 polymorphisms have been associated with severe COVID-19, it would be plausible that these polymorphisms could also exhibit an association with post-COVID symptomatology, particularly fatigue and dyspnea.

Further, these polymorphisms account for the variability of receptors’ expression of ACE2 and TMPRSS2 genes. For instance, the presence of ACE2 rs2285666 polimorphism elevates the expression of this gene up to 50%. It has been shown that higher expression of ACE2 can trigger vascular constriction, enhancing endothelial dysfunction and fibrosis as well as inflammation [24,25]. All these factors have been proposed as potential hypotheses explaining post-COVID symptoms [26]. In fact, it has been observed that plasma ACE2 activity is elevated three months after the acute infection in individuals with long-COVID as compared to uninfected matched controls [27] and that presence of long-lasting systematic inflammation after the acute phase of SARS-CoV-2 infection is associated with more severe post-COVID symptoms [28]. Accordingly, it is possible that intron/missense variants of the polymorphisms of ACE2 and TMPRSS2 genes could be associated with the develoment of post-COVID symptomatology.

Most studies investigating the role of these ACE2 or TMPRSS2 SNPs have focused on the risk of being infected or the severity of COVID-19 disease in the acute phase [3,4,5,6,7,8,9,10,11]. No study has investigated the potential association between polymorphisms of ACE2 (rs2285666 and rs2074192) and TMPRSS2 (rs12329760 and rs2070788) genes and the presence of long-term post-COVID symptoms. The aim of this study was to investigate the association between these four COVID-19 associated SNPs (ACE2 rs2285666, ACE2 rs2074192, TMPRSS2 rs12329760, TMPRSS2 rs2070788) and the presence of post-COVID symptoms in previously hospitalized COVID-19 survivors.

## 2. Methods

### 2.1. Participants

This cross-sectional study included COVID-19 survivors who had been hospitalized at three urban hospitals in Madrid (Spain) during the first wave of the pandemic (March-May 2020) because of an acute SARS-CoV-2 infection. All participants should have been diagnosed with acute SARS-CoV-2 infection by real-time reverse transcription-polymerase chain reaction (RT-PCR) assay of nasopharyngeal/oral swab sample and also presenting clinical and radiological findings at hospital admission. Patients reporting second or third re-infections were excluded. All participants provided their written informed consent prior to their inclusion. The same sample of COVID-19 survivors exhibiting post-COVID pain also participated in a previous study on pain SNPs [29], but current data presented here are new and not previously published. This study was approved by the Institutional Ethics Committees of all institutions/hospitals involved (URJC0907202015920; HUFA 20/126; HSO25112020; HUIL/092-20).

### 2.2. DNA Collection and Genotyping

Genotyping collection and management procedures had been previously published [29]. Briefly, unstimulated whole saliva samples were collected from each subject. Participants must avoid eating, drinking, chewing gum, or smoking before sample collection. All saliva samples were centrifuged at 3000 rpm for 15 min to obtain the cell sediment and stored at −20 °C.

Genomic DNA was extracted from 500 mL of saliva using a MagMAX™ DNA Multi-Sample Ultra 2.0 Kit (Thermo Fisher Scientific Inc., Hemel Hempstead, Hertfordshire, UK). We extracted DNA using the King Fisher Flex purification robot (Thermo Fisher). Purity and concentration of resulting DNA were assessed with Quant-iT™ PicoGreen™ dsDNA reagent” (Thermo Fisher). The DNA was diluted to 5 ng/μL, using 1 × Tris-EDTA (TE) buffer (Sigma-Aldrich, Dorset, UK). The qPCR reaction mixtures of 10 ul contained a total of 10 ng gDNA as a PCR template, 1× TaqMan Gene Expression PCR Master Mix, and 0.6× Genotyping TaqMan-probe assay.

The taqMan^®^ Predesigned SNP Genotyping Assays (Thermo Fisher Scientific Inc., Hertfordshire, UK) was used for genotyping the SNPs with Real-Time PCR reaction (RT-PCR). Real-time PCR plates were run in the Quantstudio 12K Flex System (Thermo Fisher) of Genomics Unit (Madrid Science Park Foundation, Madrid, Spain) under standard conditions (95° for 10 min and 40 two-step cycles consisting of 95 °C for 15 s and 60 °C for 1 min) and analyzed with Genotyping App of Thermo Fisher Cloud. Identification of each of the possible variants of each SNP was conducted by using specific fluorescent dyes.

The possible variants of the ACE2 (rs2285666) lead to an intron variant characterized by the following genotypes: C/C, C/T, T/T (C/C, C/A, A/A-C/C, C/G, G/G). The results were derived from a C→T substitution at the following sequence:

TAATCACTACTAAAAATTAGTAGC [C/T] TACCTGGTTCAAGTAATAAGCATTC

The possible variants of the ACE2 rs2074192 lead to an intron variant characterized by the following genotypes: C/C, C/T, T/T. The results were derived from a C→T substitution at the following sequence:

GTGGAAATGTATAAATGGTTGG [C/T] ATTTATTCATTTGTGACTGCTG

The possible variants of the TMPRSS2 rs12329760 lead to missense variant characterized by the following genotypes: C/C, C/T, T/T. The results were derived from a C→T substitution at the following sequence:

CTTCCTCTGAGATGAGTACA [C/T] CTGAAGGATGAAGTTTGGTC

The possible variants of the TMPRSS2 rs2070788 lead to an intron variant characterized by the following genotypes: G/G, G/A, A/A. The results were derived from a G→A substitution at the following sequence:

TGTTGTCTGTATGGCCTAGAC [G/A] CTTTTGAGAAGGATATAA

### 2.3. COVID-19 Associated Collection Data

Demographic (age, gender, height, weight), pre-existing medical comorbidities, and hospitalization (intensive care unit-ICU-admission, days at the hospital) data were collected from medical records.

Participants were scheduled for a face-to-face appointment and were asked to self-report the presence of any symptom that appeared after hospitalization due to COVID-19 and whether the symptom persisted at the time of the study. Particular attention was paid to the fact that the symptom should be attributable to COVID-19 and should have started no later than one month after SARS-CoV-2 acute infection (post-COVID-19 condition). The following list of post-COVID symptoms was created from the current literature [16,17,18] and systematically assessed: dyspnoea, fatigue, anosmia, ageusia, hair loss, pain, brain fog, ocular disorders, and loss of concentration; although participants were free to report any symptom that they suffered from and considered as relevant.

### 2.4. Statistical Analysis

Data are presented as mean (standard deviation, SD) or number of cases (percentage) as appropriate. Chi-squared (χ2) tests were conducted to assess deviations from genotype distribution from Hardy–Weinberg equilibrium. Differences in genotype frequencies of each SNP were analyzed with χ2 tests for categorical variables or one-way-ANOVA tests for continuous variables. The Shapiro–Wilk test was used to assess the assumption of normality. For all inferences, the level of significance was set at priori 0.05 with *p*-values from all tests being corrected (Holm–Bonferroni correction). Data were collected with STATA 16.1 and processed using Python’s library pandas 0.25.3. Scipy 1.5.2 program was employed for conducting the statistical tests and statsmodels 0.11.0 for performing *p*-value correction.

## 3. Results

A total of 293 (49.5% female, age: 55.6 ± 12.9 years) COVID-19 survivors from a total of 350 initially invited to participate were included. Some patients have previously been included in another of our papers [24]. Fifty-seven (16%) were excluded as follows: 1, refuse to participate (n = 30); 2, previous diagnosis of fibromyalgia (n = 20); 3, pain from neurological origin (n = 5); or, 4, pregnancy (n = 2).

Participants were assessed 17.8 ± 5.2 months after hospital discharge. At the time of the study, 257 patients (87.7%) exhibited at least one post-COVID symptom. Fatigue, pain, and memory loss were the most prevalent post-COVID symptoms with prevalence rates of 62.8%, 39.9% and 32.1%, respectively (Table 1).

From each saliva sample, DNA was isolated and amplified; however, two samples were compromised during genotyping analysis for the investigated SNPs and excluded. In addition, it was not possible to determine the genotype of ACE2 rs2074192 SNP in another sample, leading to 291 samples in ACE2 rs2285666, TMPRSS2 rs12329760, and TMPRSS2 rs2070788 genes and 290 in ACE2 rs2074192 gene.

The genotype distributions deviated from that expected based on the Hardy–Weinberg equilibrium (*p* < 0.01) which has been also extensively found in previous studies [3,4,5,6,7,8,9,10,11]. Overall, no significant differences in long-COVID symptoms were observed depending on the genotypes of any SNP: ACE2 rs2285666 (Table 2), ACE2 rs2074192 (Table 3), TMPRSS2 rs12329760 (Table 4), TMPRSS2 rs2070788 (Table 5). We only observed that the G allele of the TMPRSS2 rs2070788 was associated with higher development of post-COVID dyspnea and gastro-intestinal disorders (both, *p* < 0.01, Table 5).

There was a sex difference in the distribution of genotypes of both ACE2 (rs2285666, rs2074192) SNPs (*p* < 0.001): individuals carrying the T/C genotype in both SNPs were female (Table 2 and Table 3). No sex differences in the genotype distribution of TMPRSS2 SNPs (rs12329760, *p* = 0.604; rs2070788, *p* = 0.471) were found.

## 4. Discussion

Previous studies have investigated the associations of SNPs and the predisposition to and the severity of COVID-19 disease to identify individual risk factors [3,4,5,6,7,8,9,10,11]. It seems clear that ACE2 and TMPRSS2 receptors are important for the invasion of the SARS-CoV-2 virus into the host cell and the aggressiveness of the infection [30]. However, the role of these SNPs in the acute phase of the infection are still inconclusive with results from single studies showing an association with the severity of the infection, whereas meta-analysis have not confirmed such association [3,4,5,6,7,8,9,10,11].

Since ACE2 and TMPRSS2 polymorphisms have been potentially associated with the severity of COVID-19 condition [5,6,7,8,9,10,11], and considering that severe COVID-19 disease has been found to be associated with a higher risk of post-COVID respiratory symptoms [23], it would be reasonable to consider that these polymorphisms could be also involved in the development of post-COVID symptoms. To the best of the author knowledge, this is the first study to investigate the potential correlation between ACE2 and TMPRSS2 SNPs and the presence of long-term post-COVID symptoms in previously hospitalized COVID-19 survivors. The results did not reveal an overall association between the investigated SNPs (ACE2 rs2285666, ACE2 rs2074192, TMPRSS2 rs12329760, TMPRSS2 rs2070788) and long-COVID symptomatology suggesting that SNPs of ACE2 and TMPRSS2 genes do not predispose for developing long-COVID symptoms in previously hospitalized COVID-19 survivors. It is important to continue the research as multiple genetic variants could be important for modulating SARS-CoV-2 infection as significant variability exist across the populations [31]. Such knowledge may be also important for other viral infections and, moreover, ethnic differences should be considered to provide fundamental insight into the severe effects of the SARS-CoV-2 virus [11].

The lack of a genetic influence on long-COVID symptomatology of the investigated SNPs does not exclude the role at plasma levels of products of the ACE2 and TMPRSS2 genes in long-COVID symptoms. As it has been previously commented, plasma ACE2 activity is elevated three months after the acute infection in individuals with long-COVID [27]. In this direction, a potential role of ACE2 and TMPRSS2 products, rather than a direct effect of their polymorphisms, in the development or maintenance of long-COVID is still not fully dilucidated.

In the present study, 88% of COVID-19 survivors exhibited at least one post-COVID symptom one year and a half after the infection, reporting in line with previous studies showing fatigue, pain, and memory loss as the most prevalent symptoms [16,17,18]. The presence of post-COVID fatigue has been associated with a high burden [32], and 60% of our sample continued experiencing this symptom 18 months after hospitalization. Way to manage this symptom is crucial, since it seems to be the most prevalent and long-lasting post-COVID symptom and impact sufferers from working as efficiently as before infection [33]. In fact, some hypotheses propose that post-COVID fatigue shares common features with myalgic encephalomyelitis [34]. A common underlying pathway is based on the similar endothelial dysfunction identified in individuals with long-COVID and those with myalgic encephalomyelitis [35]. Interestingly, our study identified the G allele of the TMPRSS2 rs2070788 to be associated with the presence of post-COVID dyspnea. These results agree with previous findings showing that pulmonary TMPRSS2 expression is higher in people with GG genotype as compared to the AG and AA genotypes [36]. It is possible that some polymorphisms can be associated with specific post-COVID symptoms or that the effect of the investigated polymorphisms could induce a more severe COVID-19 phenotype, indirectly affecting the development of particular post-COVID symptoms.

It is possible that specific genes influence on particular post-COVID symptoms and genetic polymorphisms of the ACE2 might be associated with previous co-morbidities. Since ACE2 and TMPRSS2 receptors had highly expression within the lung tissue, asthma, chronic obstructive pulmonary disease, hypertension, and obesity could led to the higher expression of ACE2 [37]. Hamet et al. observed that the T allele of the SNPs rs2074192 of ACE2 gene was associated with hypertension in adult obese smoker males, but not in lean males, non-smoker males or females [38]. In our study, significant differences in previous medical co-morbidities depending on the genotype of any SNPs assessed were not found, probably due to the fact that 50% of the participants were females. In this direction, female sex has been shown to be a risk factor associated with the development of long-COVID [23], but the results of the current study found no gender associations between the SNPs of ACE2 and TMPRSS2 genes and long-COVID symptomatology.

Some limitations of this study can be discussed. First, only previously hospitalized COVID-19 survivors were included, therefore, the role of the investigated SNPs in non-hospitalized COVID-19 survivors is unknown. The inclusion of just hospitalized patients permits to determine that the lack of association between the studied SNPs and post-COVID symptoms is not related to the inclusion of patients with minimal, mild, moderate or severe COVID-19. Future studies including non-hospitalized COVID-19 survivors will help to further elucidate the role of these SNPs in post-COVID symptomatology in people with less severe COVID-19. Additionally, all patients were infected during the first wave of the outbreak, accordingly with the historical SARS-CoV-2 variant. No data about SARS-CoV-2 variants of concern and SNPs are available. Second, the potential small sample size might be not sufficient for detecting sex differences for specific genotypes. Hence, due to exploratory nature of the current study, population-based cohort studies and a whole genome SNPs analysis might help to validate the current results and identify other genes potentially related with long-COVID symptoms and co-morbidities in COVID-19 patients. Finally, this study was conducted two years after the first wave of the COVID-19 pandemic, i.e., in a period when several patients could have been vaccinated. We did not systematically collect vaccination status. A recent systematic review has summarized that the impact of vaccination in people with post-COVID symptomatology is controversial, since some studies reveal changes in symptoms whereas others did not [39]. Accordingly, we believe that the impact of vaccines on SNPs would be minimal.

## 5. Conclusions

This exploratory study showed that four polymorphisms associated with COVID-19 severity (ACE2 rs2285666, ACE2 rs2074192, TMPRSS2 rs12329760, and TMPRSS2 rs2070788), did not appear to predispose for the development of long-COVID symptoms in previously hospitalized COVID-19 survivors. Larger cohort studies investigating other polymorphisms including the analysis of sex differences could help to better understand underlying genetic pathophysiology of long-COVID and possible other relevant viral infections.

## Figures and Tables

**Table 1:** Pre-Infection data and post-COVID symptoms of the total sample.

	Total Sample (n = 293)
Age, mean (SD), years	56.5 (13.0)
Gender, female n (%)	145 (49.5%)
Weight, mean (SD), kg.	80.8 (17.0)
Height, mean (SD), cm.	168 (8.5)
Number co-morbidities, mean (SD)	1.3 (1.0)
Medical co-morbidities, n (%)	
Hypertension	103 (35.1%)
Diabetes	31 (10.6%)
Cardiovascular Diseases	21 (7.1%)
Asthma	32 (10.9%)
Obesity	90 (30.7%)
Chronic Obstructive Pulmonary Disease	6 (2.0%)
Number post-COVID symptoms, mean (SD)	3.0 (1.9)
Post-COVID symptoms, n (%)	
Fatigue	184 (62.8%)
Pain Symptoms	117 (39.9%)
Memory Loss	94 (32.1%)
Hair Loss	78 (26.6%)
Concentration Loss	44 (15.0%)
Cognitive Blunting-Brain Fog	42 (14.3%)
Dyspnoea	41 (14.0%)
Ocular Disorders	41(14.0%)
Skin Rashes	37 (12.6%)
Anosmia/Hyposmia	30 (10.2%)
Gastrointestinal Disorders	27 (9.2%)
Ageusia/Hypogeusia	21 (7.6%)
Days at hospital, mean (SD)	8.2 (7.9)

**Table 2:** Pre-Infection data and post-COVID symptoms according to ACE2 *rs2285666* Polymorphism Genotype (n = 291).

	C/C (n = 200)	C/T (n = 44)	T/T (n = 47)	*p* Value
Age, mean (SD), years	57.0 (13.0)	55.5 (13.5)	56.0 (12.0)	0.478
Gender, female n (%)	92 (46%)	42 (95.5%)	11 (23.4%)	<0.001
Weight, mean (SD), kg.	81.5 (16.5)	74.5 (14.5)	83.0 (16.5)	0.061
Height, mean (SD), cm.	168 (9.0)	162 (8.0)	170 (9.0)	0.011
Number co-morbidities, mean (SD)	1.3 (1.0)	1.3 (1.8)	1.3 (1.1)	0.988
Medical co-morbidities, n (%)				
Hypertension	75 (37.5%)	10 (22.7%)	16 (34.1%)	0.213
Diabetes	22 (11%)	3 (6.8%)	5 (10.6%)	0.724
Cardiovascular Diseases	17 (8.5%)	1 (2.3%)	1 (2.1%)	0.501
Asthma	22 (11%)	6 (13.6%)	4 (8.5%)	0.797
Obesity	59 (29.5%)	17 (38.6%)	14 (29.8%)	0.627
Chronic Obstructive Pulmonary Disease	5 (2.5%)	1 (2.3%)	0 (0%)	0.571
Number post-COVID symptoms, mean (SD)	2.9 (2.0)	3.7 (1.6)	2.6 (1.8)	0.401
Post-COVID symptoms, n (%)				
Fatigue	124 (62.0%)	33 (75.0%)	25 (53.2%)	0.429
Pain Symptoms	79 (39.5%)	20 (45.4%)	18 (38.3%)	0.735
Memory Loss	65 (32.5%)	15 (34.1%)	12 (25.5%)	0.783
Hair Loss	52 (26.0%)	16 (36.4%)	9 (19.1%)	0.314
Concentration Loss	29 (14.5%)	11 (25.0%)	4 (8.5%)	0.136
Cognitive Blunting-Brain Fog	22 (11.0%)	11 (25.0%)	8 (17.0%)	0.07
Dyspnoea	31 (15.5%)	7 (15.9%)	2 (4.25%)	0.181
Ocular Disorders	29 (14.5%)	5 (11.6%)	7 (14.9%)	0.849
Anosmia/Hyposmia	19 (9.5%)	6 (13.6%)	4 (8.5%)	0.714
Skin Rashes	26 (13.0%)	6 (13.6%)	5 (10.6%)	0.935
Gastrointestinal Disorders	20 (10.0%)	2 (4.5%)	5 (10.6%)	0.513
Ageusia/Hypogeusia	15 (7.5%)	4 (9.1%)	1 (2.1%)	0.399
Days at hospital, mean (SD)	8.3 (9.4)	7.0 (7.5)	8.5 (7.7)	0.652

**Table 3:** Pre-Infection data and post-COVID symptoms according to the ACE2 *rs2074192* Polymorphism Genotype (n = 290).

	C/C (n = 131)	C/T (n = 66)	T/T (n = 93)	*p* Value
Age, mean (SD), years	56.1 (13.0)	56.0 (13.0)	57.5 (12.5)	0.604
Gender, female n (%)	49 (37.4%)	65 (98.5%)	31 (33.3%)	<0.001
Weight, mean (SD), kg.	83.1 (16.3)	74.5 (16.3)	82.3 (16.4)	0.001
Height, mean (SD), cm.	169 (9.5)	164 (7.5)	168 (9.0)	0.001
Number co-morbidities, mean (SD)	1.4 (1.1)	1.3 (0.9)	1.2 (0.9)	0.489
Medical co-morbidities, n (%)				
Hypertension	47 (35.9%)	20 (30.3%)	34 (36.5%)	0.647
Diabetes	9 (6.6%)	7 (10.6%)	14 (15.0%)	0.184
Cardiovascular Diseases	12 (9.1%)	2 (3.0%)	7 (7.5%)	0.306
Asthma	16 (12.9%)	9 (13.6%)	7 (7.5%)	0.154
Obesity	47 (35.9%)	20 (30.3%)	23 (24.7%)	0.302
Chronic Obstructive Pulmonary Disease	2 (1.5%)	0 (0.0%)	4 (4.3%)	0.153
Number post-COVID symptoms, mean (SD)	2.8 (1.9)	3.3 (1.9)	2.9 (1.9)	0.222
Post-COVID symptoms, n (%)				
Fatigue	79 (60.3%)	45 (68.2%)	58 (62.4%)	0.811
Pain Symptoms	52 (39.7%)	34 (51.5%)	30 (32.2%)	0.045
Memory Loss	34 (25.9%)	21 (31.8%)	37 (39.8%)	0.217
Hair Loss	26 (19.8%)	26 (39.4%)	25 (26.0%)	0.06
Concentration Loss	22 (16.8%)	12 (18.2%)	10 (10.7%)	0.391
Cognitive Blunting-Brain Fog	17 (13.0%)	14 (21.2%)	10 (10.7%)	0.207
Dyspnoea	15 (11.4%)	10 (15.1%)	15 (16.1%)	0.641
Ocular Disorders	19 (14.5%)	10 (15.1%)	12(12.9%)	0.915
Anosmia/Hyposmia	12 (9.2%)	6 (9.1%)	11 (11.8%)	0.809
Skin Rashes	13 (9.9%)	10 (15.1%)	14 (15.1%)	0.498
Gastrointestinal Disorders	16 (12.2%)	5 (7.6%)	6 (6.5%)	0.310
Ageusia/Hypogeusia	9 (6.9%)	6 (9.1%)	5 (5.4%)	0.681
Days at hospital, mean (SD)	7.5 (6.5)	7.2 (6.8)	9.8 (12.2)	0.08

**Table 4:** Pre-Infection data and post-COVID symptoms according to the TMPRSS2 *rs12329760* Polymorphism Genotype (n = 291).

	C/C (n = 223)	C/T (n = 64)	T/T (n = 4)	*p* Value
Age, mean (SD), years	56.0 (12.5)	58.5 (13.0)	60.0 (10.5)	0.251
Gender, female n (%)	105 (47.1%)	37 (57.8%)	2 (50.0%)	0.604
Weight, mean (SD), kg.	81.0 (16.5)	80.0 (16.0)	89.2 (14.0)	0.545
Height, mean (SD), cm.	167 (9.5)	168 (8.5)	171 (10.0)	0.682
Number co-morbidities, mean (SD)	1.3 (1.0)	1.3 (1.0)	1.5 (1.3)	0.911
Medical co-morbidities, n (%)				
Hypertension	76 (34.1%)	23 (35.9%)	1 (25.0%)	0.933
Diabetes	20 (8.9%)	9 (14.1%)	1 (25.0%)	0.367
Cardiovascular Diseases	17 (7.6%)	4 (6.25%)	0 (0.0%)	0.801
Asthma	25 (11.2%)	6 (9.4%)	1 (25.0%)	0.643
Obesity	73 (32.7%)	15 (23.4%)	2 (50.0%)	0.375
Chronic Obstructive Pulmonary Disease	4 (1.8%)	2 (3.1%)	0 (0.0%)	0.782
Number post-COVID symptoms, mean (SD)	3.0 (1.9)	3.0 (1.9)	3.25 (2.5)	0.907
Post-COVID symptoms, n (%)				
Fatigue	137 (61.4%)	44 (68.7%)	2 (50.0%)	0.764
Pain Symptoms	92 (41.2%)	22 (34.7%)	2 (50.0%)	0.221
Memory Loss	70 (31.4%)	20 (31.2%)	2 (50.0%)	0.811
Hair Loss	63 (28.2%)	13 (20.3%)	1 (25.0%)	0.525
Concentration Loss	33 (14.8%)	8 (12.5%)	3 (75.0%)	0.214
Cognitive Blunting-Brain Fog	32 (14.3%)	8 (12.5%)	1 (25.0%)	0.788
Dyspnoea	29 (13.0%)	11 (17.2%)	0 (0.0%)	0.566
Ocular Disorders	28 (12.5%)	12 (18.7%)	1 (25.0%)	0.451
Anosmia/Hyposmia	21 (9.4%)	8 (12.5%)	0 (0.0%)	0.658
Skin Rashes	32 (14.3%)	5 (7.8%)	0 (0.0%)	0.321
Gastrointestinal Disorders	19 (8.5%)	8 (12.5%)	0 (0.0%)	0.502
Ageusia/Hypogeusia	13 (5.8%)	6 (9.4%)	1 (25.0%)	0.251
Days at hospital, mean (SD)	8.6 (9.3)	6.5 (5.6)	9.25 (8.3)	0.245

**Table 5:** Pre-Infection data and post-COVID symptoms according to the TMPRSS2 *rs2070788* Polymorphism Genotype (n = 291).

	A/A (n = 79)	A/G (n = 144)	G/G (n = 68)	*p* Value
Age, mean (SD), years	56.5 (13.0)	57.0 (12.5)	56.0 (13.0)	0.829
Gender, female n (%)	34 (43.1%)	71 (49.3%)	39 (57.3%)	0.471
Weight, mean (SD), kg.	80.8 (15.5)	80.3 (16.0)	82.1 (19.5)	0.773
Height, mean (SD), cm.	169 (8.9)	167 (9.5)	166 (10.3)	0.501
Number co-morbidities, mean (SD)	1.3 (1.0)	1.3 (1.0)	1.3 (1.0)	0.972
Medical co-morbidities, n (%)				
Hypertension	24 (30.4%)	51 (35.4%)	25 (36.8%)	0.742
Diabetes	12 (15.2%)	10 (6.9%)	8 (11.8%)	0.188
Cardiovascular Diseases	7 (8.9%)	8 (5.5%)	6 (8.8%)	0.609
Asthma	9 (11.4%)	18 (12.5%)	5 (7.3%)	0.544
Obesity	23 (29.1%)	46 (31.9%)	21 (30.9%)	0.903
Chronic Obstructive Pulmonary Disease	1 (1.3%)	3 (2.1%)	2 (3.0%)	0.780
Number post-COVID symptoms, mean (SD)	2.9 (2.0)	3.0 (1.9)	3.1 (1.8)	0.728
Post-COVID symptoms, n (%)				
Fatigue	49 (62.0%)	91 (63.2%)	41 (60.3%)	0.931
Pain Symptoms	30 (37.9%)	56 (38.9%)	31 (45.6%)	0.581
Memory Loss	23 (29.1%)	45 (31.2%)	24 (35.3%)	0.803
Hair Loss	19 (24.0%)	39 (27.1%)	19 (27.9%)	0.862
Concentration Loss	13 (16.5%)	21 (14.6%)	10 (14.7%)	0.951
Cognitive Blunting-Brain Fog	13 (16.5%)	19 (13.2%)	9 (13.2%)	0.827
Dyspnoea	7 (8.9%)	17 (11.8%)	16 (23.5%)	0.023
Ocular Disorders	12 (15.2%)	20 (13.9%)	9 (13.2%)	0.951
Anosmia/Hyposmia	7 (8.9%)	17 (11.8%)	5 (7.3%)	0.558
Skin Rashes	13 (16.5%)	12 (8.3%)	12 (17.6%)	0.129
Gastrointestinal Disorders	6 (7.6%)	9 (6.2%)	12 (17.6%)	0.01
Ageusia/Hypogeusia	7 (8.9%)	9 (6.2%)	4 (5.9%)	0.743
Days at hospital, mean (SD)	7.7 (7.0)	8.8 (10.5)	7.5 (7.0)	0.545

## Data Availability

All data derived from this study are presented in the text.
